# Supplementary material for: Catalytic pocket of Clr4 (Suv39h) methyltransferase serves as a substrate receptor for Cullin 4-dependent histone H3 ubiquitination
Source: bioRxiv. 2025 Aug 30:2025.08.28.672867. Preprint. [Version 1] doi: 10.1101/2025.08.28.672867 (PMC12407891; doi:10.1101/2025.08.28.672867)
Supplement: Supplement 1 [file NIHPP2025.08.28.672867v1-supplement-1.pdf]

bioRxiv preprint doi: https://doi.org/10.1101/2025.08.28.672867; this version posted August 30, 2025. The copyright holder for this preprint (which was not certified by peer review) is the author/funder, who has granted bioRxiv a license to display the preprint in perpetuity. It is made available under aCC-BY 4.0 International license.

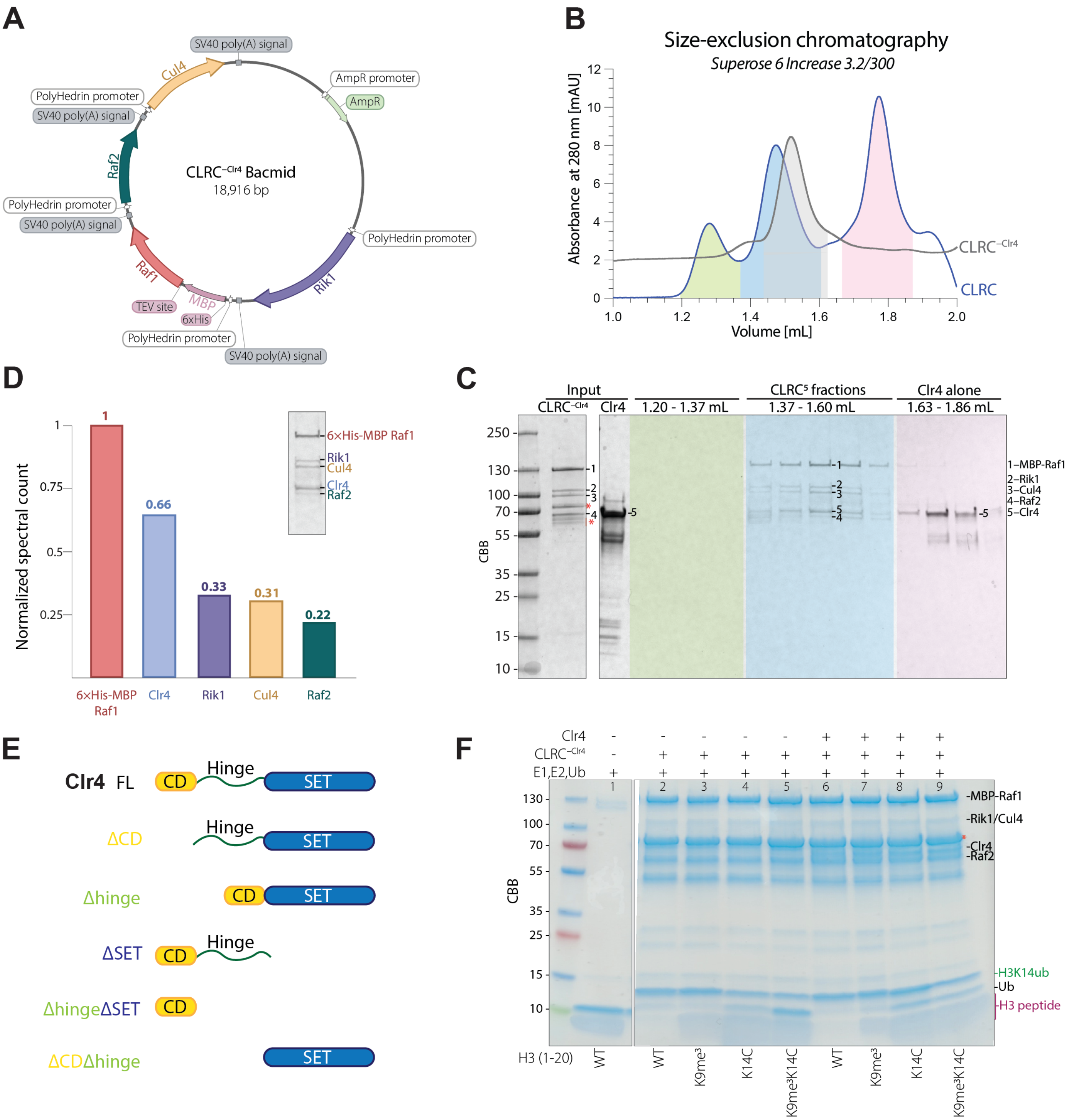

Figure S1: Purification of the CLRC complex. (A) Map of Recombinant Bacmid Vector Encoding CLRC-Clr4. Diagram of the recombinant bacmid vector used for insect cell expression, encoding all CLRC components except Clr4. Key vector features and regulatory elements are indicated. **(B)** SEC chromatogram of CLRC complexes using Superose 6 Increase 3.2/300 column (Cytiva). Absorbance at 280 nm (mAU) is shown. The grey line indicated the elution profile of CLRC complex lacking Clr4, with the peak fractions highlighted. The blue line indicated the elution profile of the full CLRC complex with peak fractions highlighted in blue and fractions containing excess unbound Clr4 highlighted in pink. **(C)** SDS-PAGE analysis of SEC fractions from panel B. Coomassie-stained SDS–PAGE gel showing input (CLRC-Clr4 and Clr4) and eluted fractions from size-exclusion chromatography. Fractions corresponding to the blue peak (1.45–1.55 mL) contain all 5 CLRC complex subunits, while later fractions highlighted in pink (1.65–1.85 mL) contain excess unbound Clr4. *, MBP-Raf1 degradation products. **(D)** Mass spectrometry analysis of the reconstituted CLRC complex. Quantitative LC-MS/MS analysis of the reconstituted CLRC complex showing normalized spectral counts relative to MBP–Raf1 (set to 1). The calculated molar ratios of 0.66 for Clr4, 0.33 for Rik1, 0.31 for Cul4, and 0.22 for Raf2 are consistent with SDS–PAGE band intensities. **(E)** Schematic representation of Clr4 truncation constructs used in this study. Domain architecture of Clr4 and its truncation variants, with the chromodomain shown in yellow, the hinge region in green, and the SET domain in blue. **(F)** SDS-PAGE analysis of in vitro H3 ubiquitination by reconstituted CLRC complex. Ubiquitination reactions were performed using recombinant human E1, E2, HA–ubiquitin (Ub), and the N-terminal peptide of histone H3 (residues 1–20), in the presence of reconstituted CLRC complexes with or without Clr4. Both CLRC and CLRC-Clr4 efficiently ubiquitinated the H3 peptide, whereas E1 and E2 alone showed no activity. *, MBP-Raf1 degradation products; HO, histone octamer.

bioRxiv preprint doi: https://doi.org/10.1101/2025.08.28.672867; this version posted August 30, 2025. The copyright holder for this preprint (which was not certified by peer review) is the author/funder, who has granted bioRxiv a license to display the preprint in perpetuity. It is made available under aCC-BY 4.0 International license.

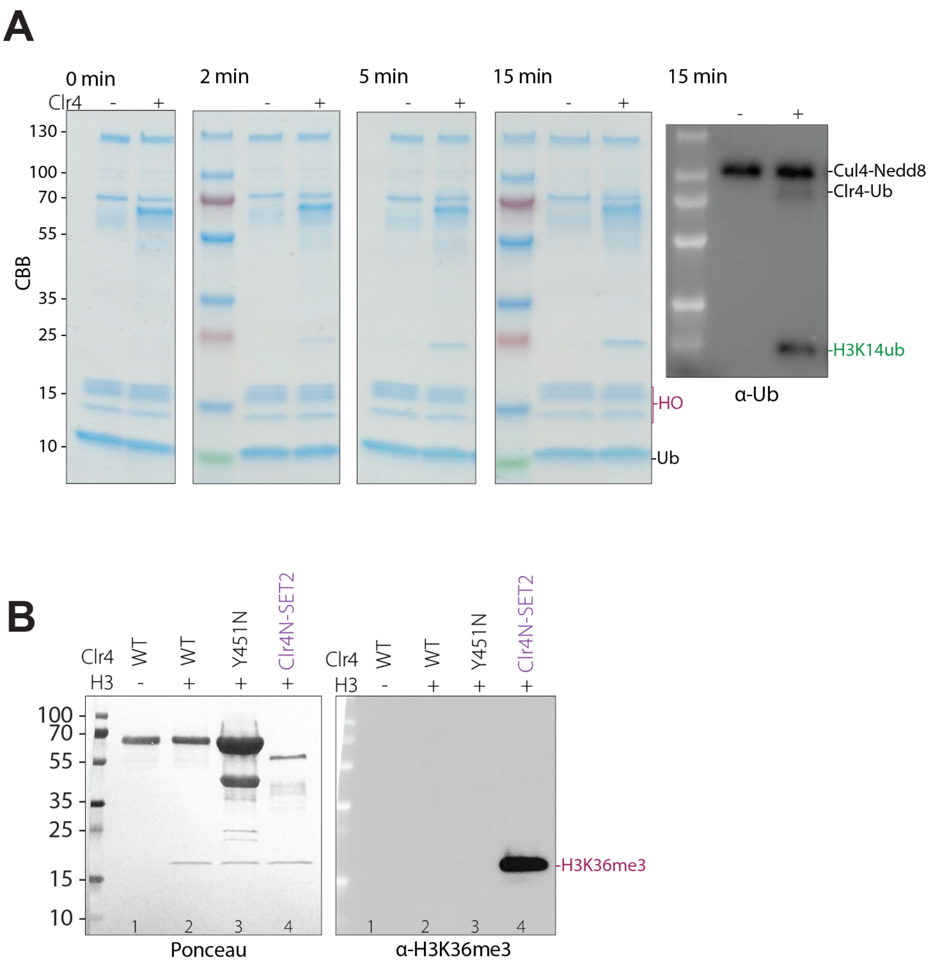

Figure S2: The kinetics of H3K14 ubiquitination and its Clr4-dependence. (A) SDS–PAGE analysis of time-course ubiquitination of nucleosome by reconstituted CLRC ± Clr4 complex. Reactions were sampled at the indicated time points to monitor the progression of nucleosome ubiquitination. HO, histone octamer. **(B)** Clr4N-Set2 fusion protein retains H3K36 methylation specificity. Immunoblotting with anti-H3K36me3 antibody demonstrating that the Clr4N-Set2 fusion methylated H3K36.

bioRxiv preprint doi: https://doi.org/10.1101/2025.08.28.672867; this version posted August 30, 2025. The copyright holder for this preprint (which was not certified by peer review) is the author/funder, who has granted bioRxiv a license to display the preprint in perpetuity. It is made available under aCC-BY 4.0 International license.

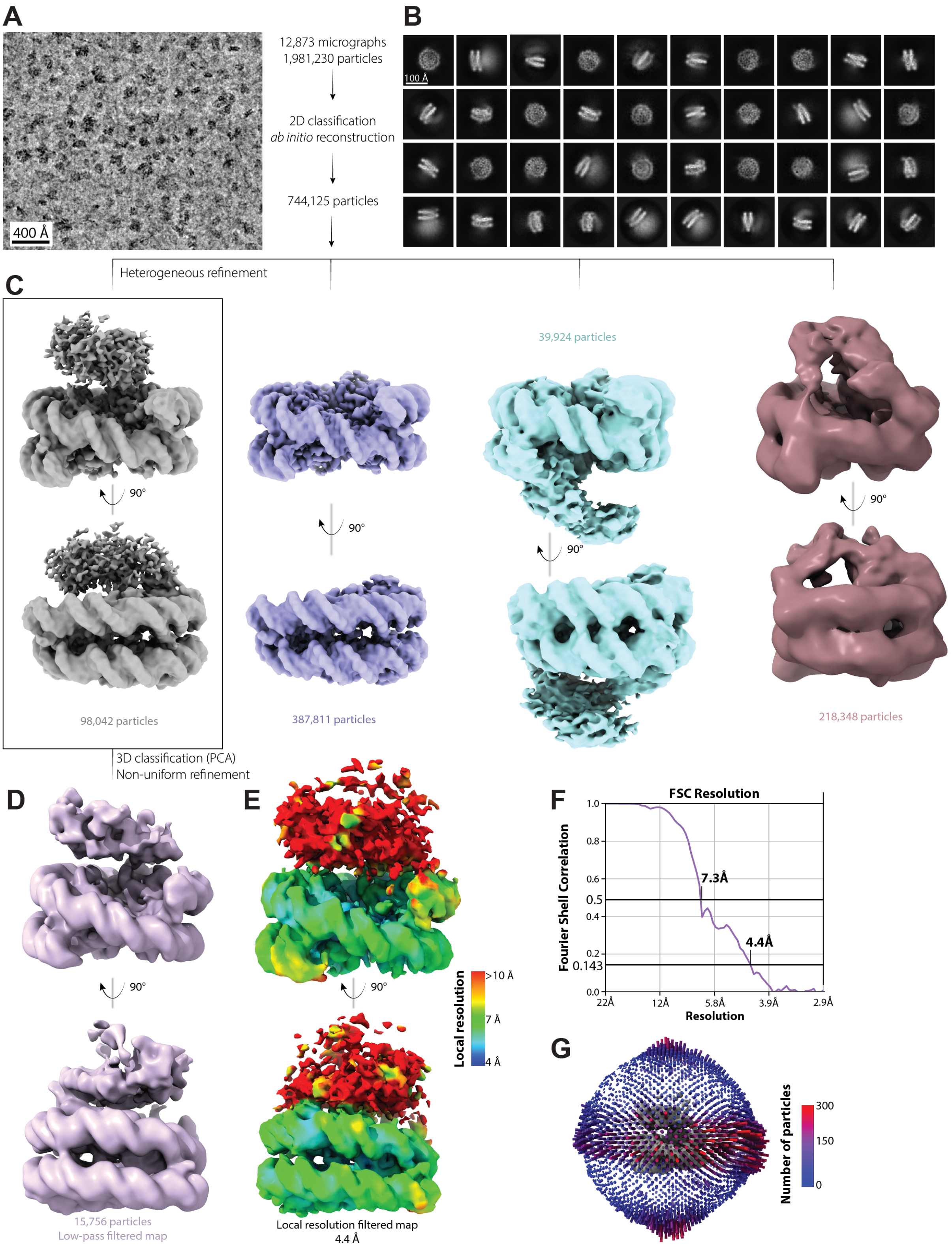

Figure S3: CryoEM data acquisition and processing workflow. Cryo-EM data were collected on a Titan Krios (300 keV) with a Gatan K3 detector. **(A)** Representative low-pass filtered micrograph (upper left; scale bar 400 Å) and classification tree show monodisperse particles. Of 13,149 micrographs, 12,873 were retained after curation. Image processing in CryoSPARC v4.4.1 included blob picking (1,981,230 particles), local motion correction (280-pixel box), and 2D classification. **(B)** Representative 2D class averages (scale bar 100 Å). **(C)** A total of 744,125 particles were used for ab initio reconstruction and multiple rounds of heterogeneous refinement. The map (gray) used for further processing is highlighted by a rectangle. The gray map showed best agreement with the HADDOCK derived model. **(D)** The final low-pass filtered map used for model building, comprising 15,756 particles, was selected from 3D classification based on principal component analysis (PCA). **(E)**The final map was further processed using non-uniform refinement with local resolution filtering. The overall resolution, estimated by FSC, was 4.4 Å. Local resolution analysis (colored from red [low] to blue [high]) revealed that the additional density was of substantially lower resolution, with the global resolution value largely reflecting the high-resolution reconstruction of the nucleosome core. **(F)** FSC curve of the final map from panel E. **(G)** Angular particle distribution plot of the final map from panel E.

bioRxiv preprint doi: https://doi.org/10.1101/2025.08.28.672867; this version posted August 30, 2025. The copyright holder for this preprint (which was not certified by peer review) is the author/funder, who has granted bioRxiv a license to display the preprint in perpetuity. It is made available under aCC-BY 4.0 International license.

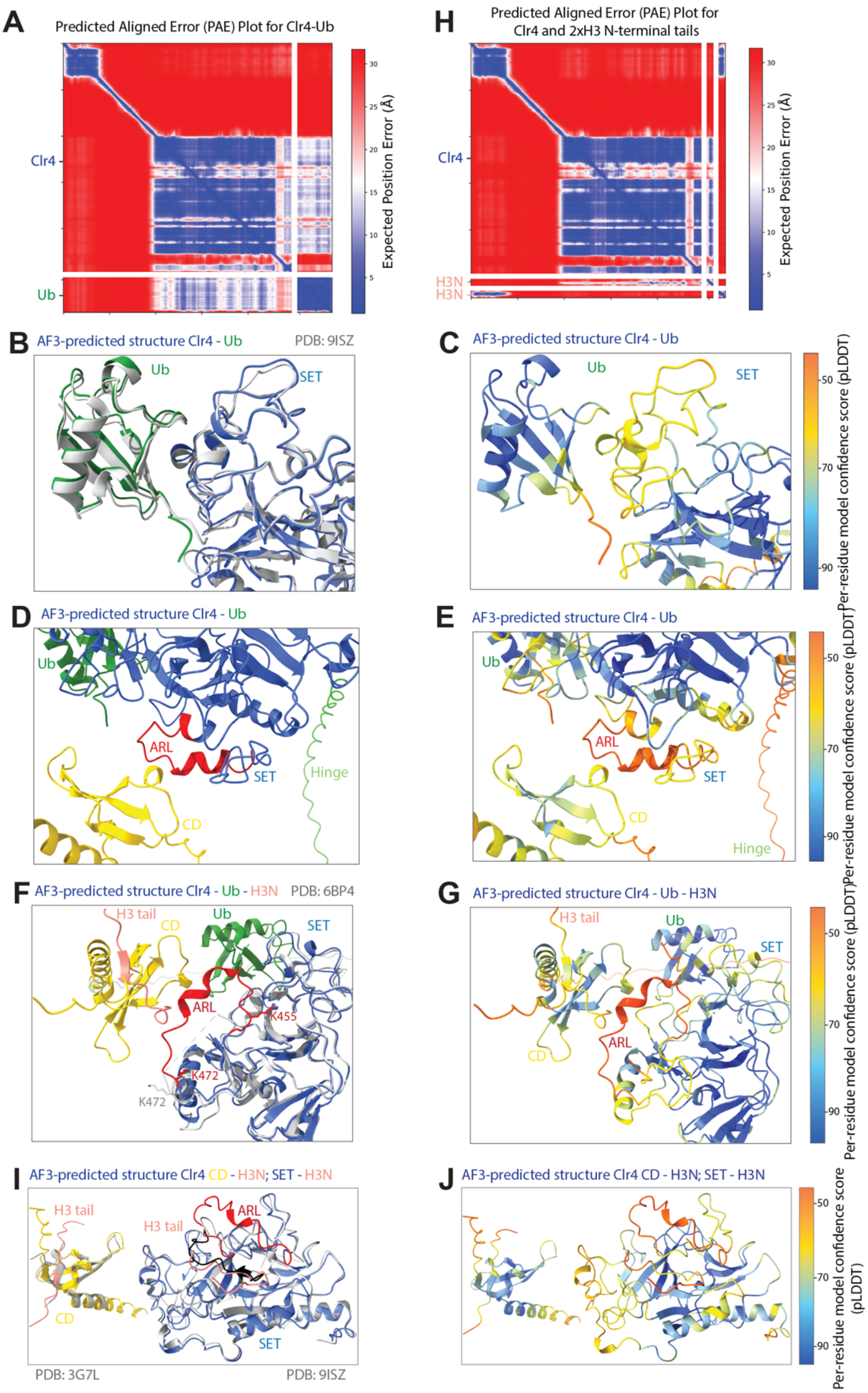

Figure S4: AlphaFold3-based structural predictions of Clr4 and its interactions with ubiquitin and histone H3 tails. (A) Predicted Aligned Error (PAE) plots for AlphaFold3 (AF3) model of Clr4-ubiquitin. PAE plot show the predicted alignment error between residue pairs for the Clr4-ubiquitin complex. Low predicted error (blue) indicates high confidence in residue placement; high error (white/red) reflects flexibility or lack of interaction. **(B)** Overlay of the AF3-predicted Clr4–ubiquitin complex with the crystal structure of Clr4-SET bound to an H3K14ub peptide. The predicted interface between the Clr4 SET domain (blue) and ubiquitin (green) closely matches the crystal structure (PDB 9ISZ, grey). The corresponding PAE plot is shown in panel A. **(C)** Per-residue model confidence (pLDDT) for the AF3 prediction in panel B. High-confidence regions are indicated in blue; lower-confidence regions are shown in green to red. **(D)** AF3-predicted intramolecular interaction between Clr4's autoregulatory loop (ARL, red) and chromodomain (CD, yellow). **(E)** pLDDT scores for the predicted structure in panel D. **(F, G)** Overlay of the predicted Clr4 SET domain structure with the crystal structure of open automethylated Clr4 SET domain. The AF3-predicted structure (blue) aligns closely with the SET domain in its open conformation (PDB 6BP4, grey). pLDDT confidence scores are shown in panel G and the PAE plot in panel A. **(H)** Predicted Aligned Error (PAE) plots for AlphaFold3 (AF3) models of Clr4-H3 tail. **(I, J)** Overlay of the AF3-predicted interactions between Clr4 and H3 tails with experimental structures. AF3-predicted binding of the Clr4 CD (yellow) and SET domain (blue) to the H3 N-terminal tail (salmon) is in close agreement with

bioRxiv preprint doi: https://doi.org/10.1101/2025.08.28.672867; this version posted August 30, 2025. The copyright holder for this preprint (which was not certified by peer review) is the author/funder, who has granted bioRxiv a license to display the preprint in perpetuity. It is made available under aCC-BY 4.0 International license.

respective crystal structures of CD-H3 (PDB 3G7L, grey) and SET–H3 (PDB 9ISZ, grey) (I). pLDDT confidence scores are shown in panel J and the PAE plot in panel H.

bioRxiv preprint doi: https://doi.org/10.1101/2025.08.28.672867; this version posted August 30, 2025. The copyright holder for this preprint (which was not certified by peer review) is the author/funder, who has granted bioRxiv a license to display the preprint in perpetuity. It is made available under aCC-BY 4.0 International license.

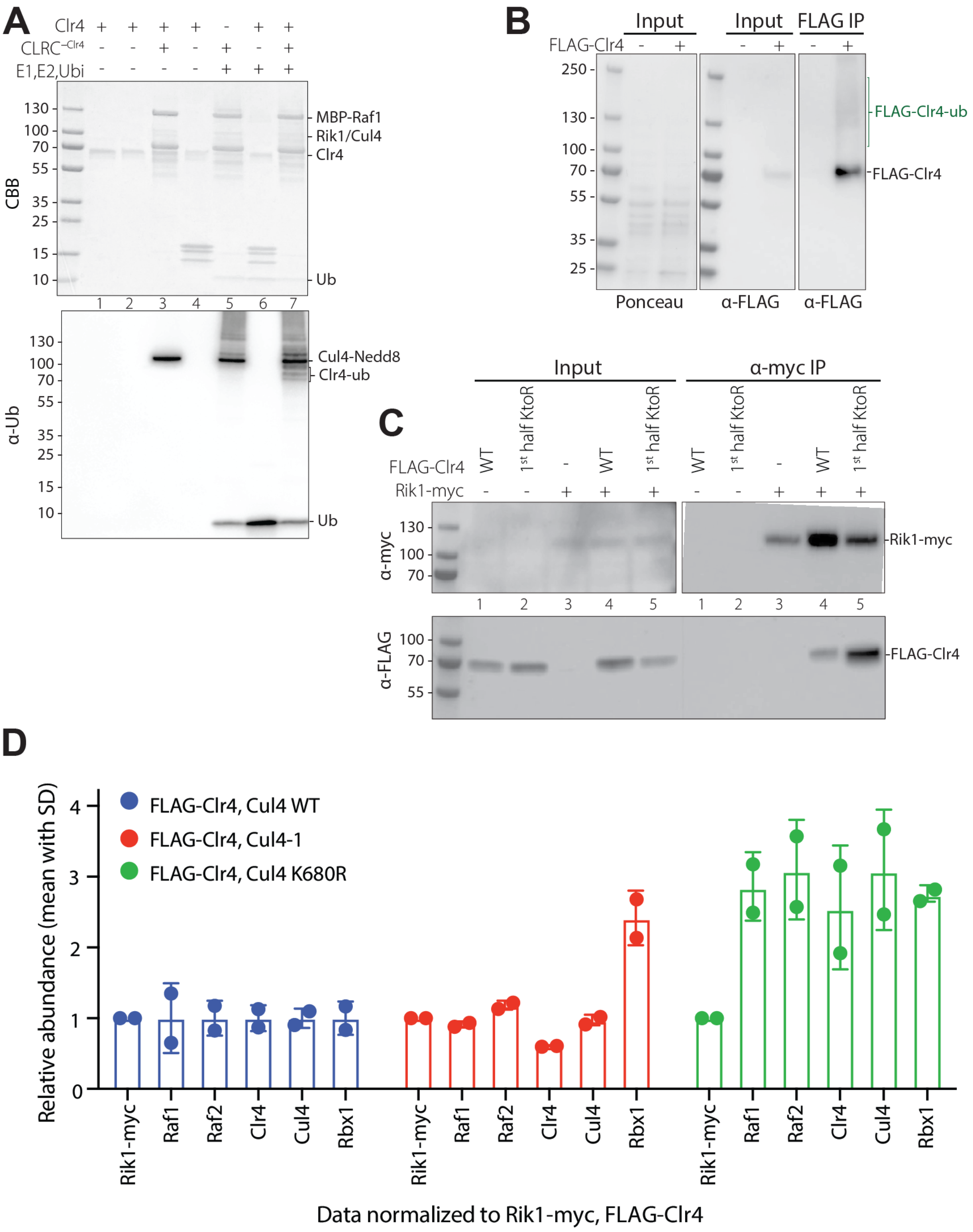

Figure S5: CLRC mediated Clr4 ubiquitination and release of Clr4 from the CLRC complex. (A) CLRC-dependent ubiquitination of Clr4 in vitro. Anti-ubiquitin western blot of in vitro reactions using purified CLRC complexes. Mono- and di-ubiquitinated Clr4 species (lane 7) appeared only in the presence of the full CLRC complex, but not with E1/E2 enzymes or CLRC lacking Clr4 (lanes 5–6), indicating CLRC-dependent ubiquitination. The anti-ubiquitin antibody also cross-reacts with Nedd8, resulting in a detectable Cul4-Nedd8 band. **(B)** Detection of polyubiquitinated Clr4 species in vivo. Western blot analysis of FLAG-Clr4 purified from *S. pombe* in the presence of the proteasome inhibitor MG-132 and deubiquitinase inhibitor NEM revealed slower-migrating bands, consistent with polyubiquitinated forms of Clr4. **(C)** Loss of Clr4 ubiquitination enhances interaction with CLRC. Western blot analysis of co-immunoprecipitation experiments in *S. pombe* strains expressing myc-tagged Rik1 and FLAG-Clr4 variants. Immunoprecipitation with anti-myc beads revealed that Clr4 1st half KtoR mutants, which lack ubiquitination in the chromodomain and hinge regions, exhibited increased association with the CLRC complex compared to wild-type Clr4. **(D)** Quantitative proteomic analysis of CLRC complex assembly in *cul4* mutant backgrounds. TMT-based mass spectrometry of FLAG-Clr4 immunoprecipitates from *S. pombe* cells expressing wild-type *cul4*, *cul4-1*, or *cul4-K680R*. In *cul4-K680R* mutant cells, FLAG-Clr4 showed ~3-fold increased association with other CLRC subunits, indicating impaired dis-assembly. By contrast, the *cul4-1* hypomorphic allele reduced Clr4 association with Rik1 and overall CLRC assembly, consistent with decreased ubiquitination activity.

bioRxiv preprint doi: https://doi.org/10.1101/2025.08.28.672867; this version posted August 30, 2025. The copyright holder for this preprint (which was not certified by peer review) is the author/funder, who has granted bioRxiv a license to display the preprint in perpetuity. It is made available under aCC-BY 4.0 International license.

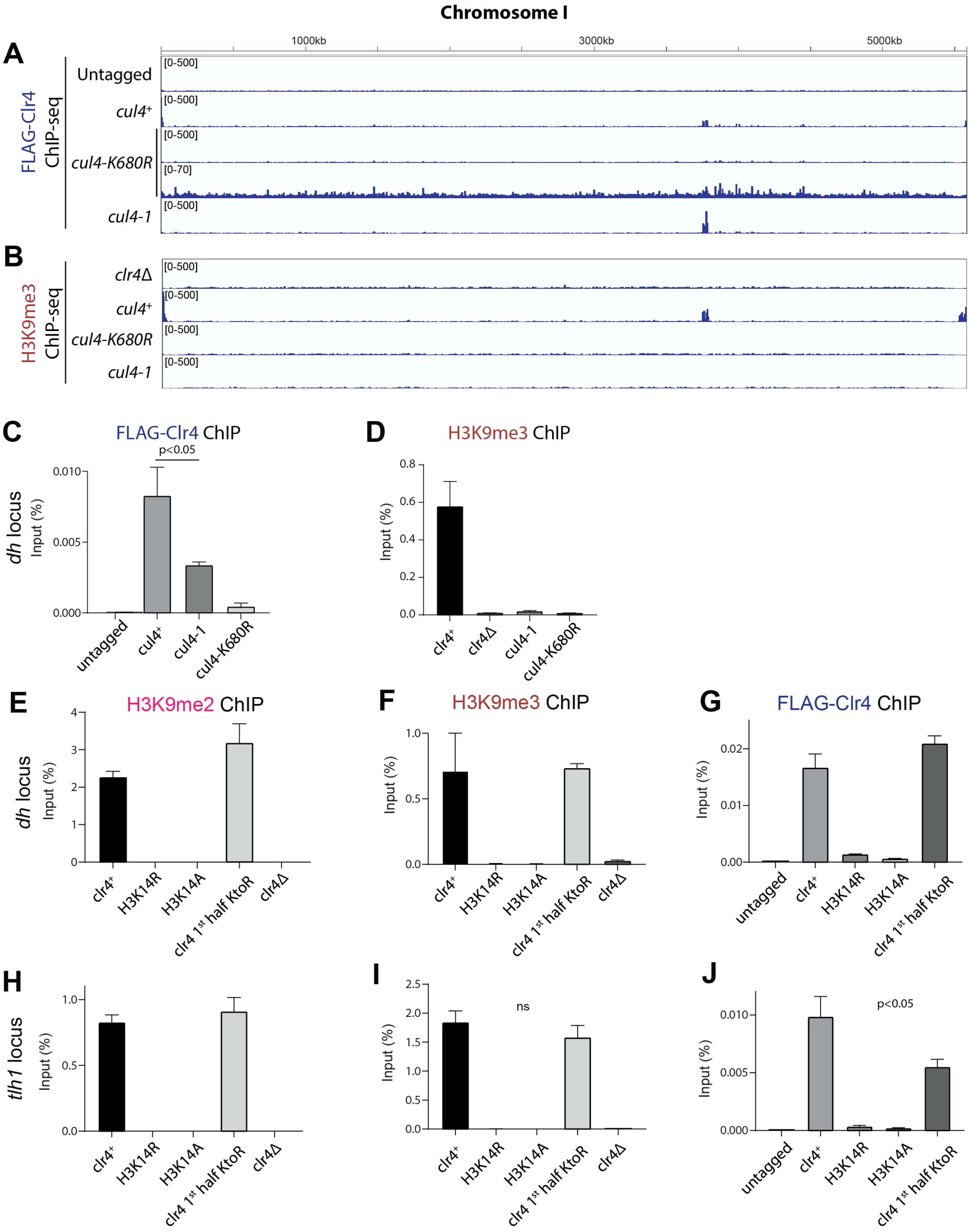

Figure S6.:Role of Cul4-mediated ubiquitination in Clr4 localization and H3K9 methylation. Related to Figure 6. (A, B) Genome browser views of ChIP-seq data showing the localization of FLAG-Clr4 (A) and H3K9me3 (B) along the *S. pombe* chromosome I in wild-type (*cul4+*), *cul4-K680R*, and *cul4-1* cells. **(C-J)** ChIP-qPCR analysis of FLAG-Clr4 and H3K9me3 in wild-type, *cul4-K680R*, *cul4-1*, *H3K14R*, *H3K14A*, *1st half-KtoR* (clr4 with 1st half lysines substituted with arginine, see Figure 5B), and *clr4Δ* cells at the indicated loci. Bars show mean percentage input and error bars show standard deviations of 3 biological replicates.

bioRxiv preprint doi: https://doi.org/10.1101/2025.08.28.672867; this version posted August 30, 2025. The copyright holder for this preprint (which was not certified by peer review) is the author/funder, who has granted bioRxiv a license to display the preprint in perpetuity. It is made available under aCC-BY 4.0 International license.

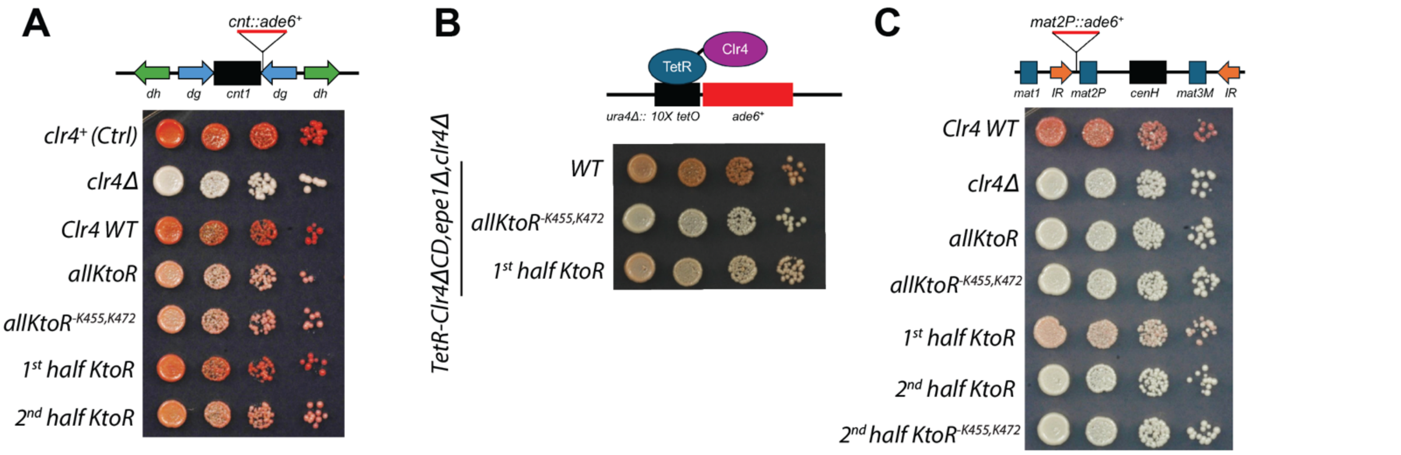

Figure S7: Role of Cul4-mediated ubiquitination in silencing. (**A**) Silencing assays for a centromeric *ade6*+ reporter (*otr1R::ade6*+). Ten-fold serial dilutions of cells were plated on low adenine YE plates in which silencing of *ade6*+ leads to formation of red/pink colonies. (**B**) Silencing assays for the *ade6*+ reporter inserted at the mating type locus (*mat2P::ade6*+). Cells were plated as in panel A. **(C)** Silencing assays for the *ura4Δ::10XtetO-ade6*+ reporter cells in which the recruitment of TetR-Clr4-ΔCD initiator leads to formation of heterochromatin and *ade6*+ silencing. Wild-type (WT) refers to TetR-Clr4-ΔCD without any amino acid substitutions.

bioRxiv preprint doi: https://doi.org/10.1101/2025.08.28.672867; this version posted August 30, 2025. The copyright holder for this preprint (which was not certified by peer review) is the author/funder, who has granted bioRxiv a license to display the preprint in perpetuity. It is made available under aCC-BY 4.0 International license.

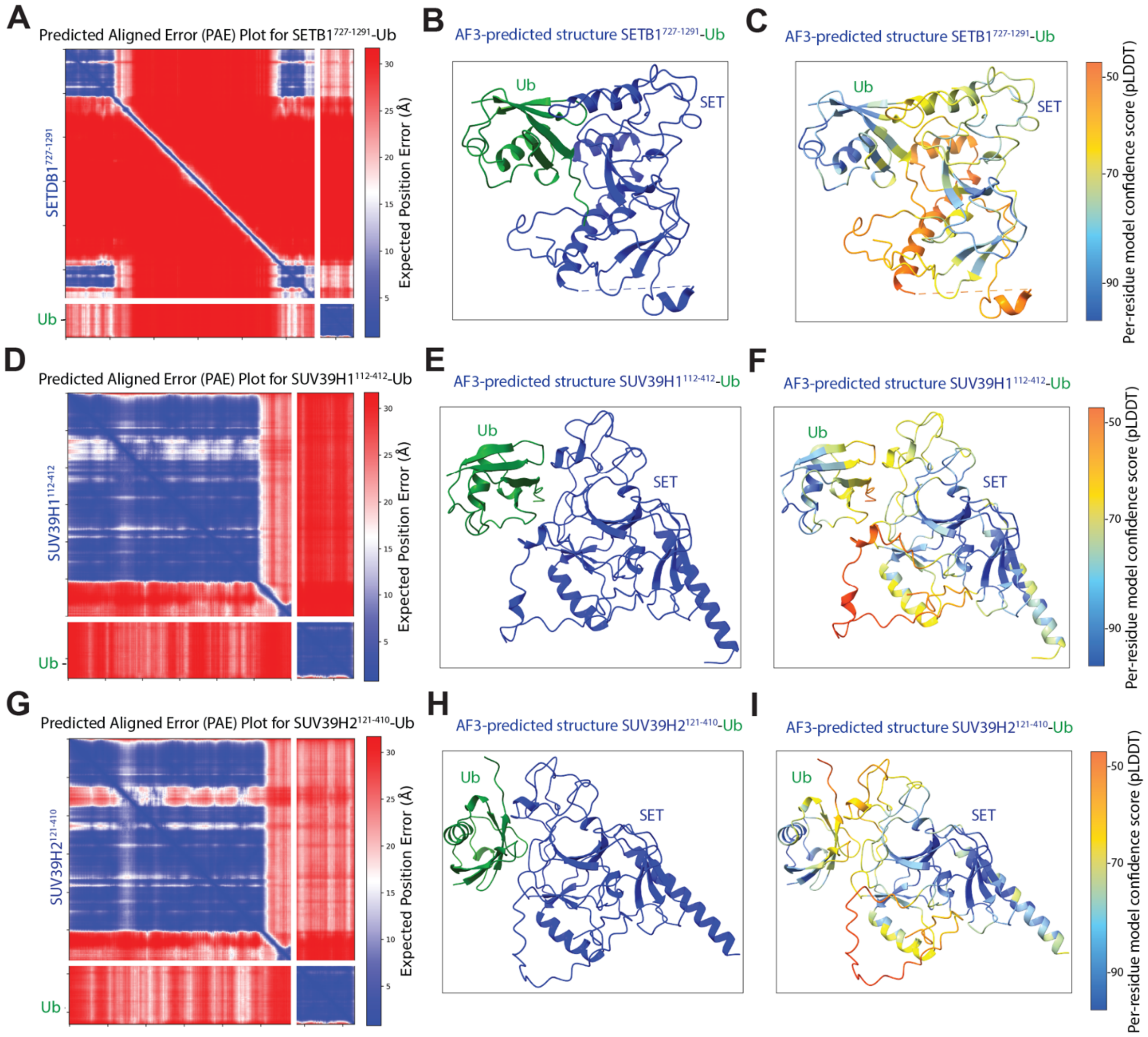

Figure S8: AlphaFold3 structural predictions of the interactions of SUV39 subfamily of SET-domain-containing histone methyltransferases with ubiquitin. (A, D, G) Predicted Aligned Error (PAE) plots for AlphaFold3 (AF3) models of the indicated SET domains and ubiquitin (Ub). PAE plots show the predicted alignment error between residue pairs for the SETDB1$^{727\text{-}1291}$-ubiquitin complex (A), SUV39H1$^{112\text{-}412}$-ubiquitin complex (D), SUV39H2$^{121\text{-}410}$-ubiquitin complex (G). Low predicted error (blue) indicates high confidence in residue placement; high error (white/red) reflects flexibility or lack of predicted interaction. **(B, E, H)** AF3-predicted interactions between SUV39 subfamily of SET-domain-containing histone methyltransferases and ubiquitin. The SET domains are highlighted in blue, ubiquitin in green. **(C, F, I)** Per-residue model confidence (pLDDT) for the AF3 predictions in panels B, E, H. High-confidence regions are indicated in blue; lower-confidence regions are shown in green to red.

bioRxiv preprint doi: https://doi.org/10.1101/2025.08.28.672867; this version posted August 30, 2025. The copyright holder for this preprint (which was not certified by peer review) is the author/funder, who has granted bioRxiv a license to display the preprint in perpetuity. It is made available under aCC-BY 4.0 International license.

Table S1: Summary of inter- and intra-Clr4 cross-links identified by BS3 XL-MS. All cross-links detected with Clr4 using the amine-reactive cross-linker BS3, which links lysine side chains or N-terminal α-amino groups. The BS3 spacer arm, together with lysine side chains, allows for cross-links up to approximately 30 Å in Cα-Cα distance. To assess structural compatibility, each cross-link was mapped onto the structural model and color-coded by the measured Cα-Cα distance: green: distances ≤ 30 Å, considered compatible with cross-linking constraints; pink: distances between 31-34 Å, likely reflecting flexible regions; dark red: distances > 35 Å, exceeding the cross-linker limit and indicating possible violations or conformational variability. Structural domains and regions are annotated as follows: chromodomain (CD) in yellow, hinge region in light green, SET domain in blue, ARL in red, ubiquitin (Ub) in dark green, and H3 N-terminal tail in purple. All identified crosslinks, including those within the nucleosome, are reported in Table S5.

Protein #1	Amino acid #1	Residue number #1	Protein #2	Amino acid #2	Residue number #2	Crosslinked regions	Distance
Clr4	GLY	1	Clr4	LYS	17	CD-CD	17 Å
Clr4	GLY	1	Clr4	LYS	25	CD-CD	18 Å
Clr4	GLY	1	Clr4	LYS	94	CD-hinge	26 Å
Clr4	GLY	1	Clr4	LYS	109	CD-hinge	29 Å
Clr4	GLY	1	Clr4	LYS	114	CD-hinge	27 Å
Clr4	GLY	1	Clr4	LYS	122	CD-hinge	20 Å
Clr4	GLY	1	Clr4	LYS	187	CD-hinge	39 Å
Clr4	GLY	1	Clr4	LYS	472	CD-ARL	24 Å
Clr4	LYS	17	Clr4	LYS	25	CD-CD	6 Å
Clr4	LYS	17	Clr4	LYS	472	CD-ARL	24 Å
Clr4	LYS	109	Clr4	LYS	114	Hinge-hinge	11 Å
Clr4	LYS	122	Clr4	LYS	160	Hinge-hinge	18 Å
Clr4	LYS	151	Clr4	LYS	160	Hinge-hinge	16 Å
Clr4	LYS	151	Clr4	LYS	165	Hinge-hinge	25 Å
Clr4	LYS	154	Clr4	LYS	165	Hinge-hinge	18 Å
Clr4	LYS	160	Clr4	LYS	174	Hinge-hinge	25 Å
Clr4	LYS	165	Clr4	LYS	174	Hinge-hinge	14 Å
Clr4	LYS	165	Clr4	LYS	472	Hinge-ARL	23 Å
Clr4	LYS	174	Clr4	LYS	187	Hinge-hinge	6 Å
Clr4	LYS	174	Clr4	LYS	472	Hinge-ARL	20 Å
Clr4	LYS	180	Clr4	LYS	193	Hinge-SET	24 Å
Clr4	LYS	187	Clr4	LYS	193	Hinge-SET	17 Å
Clr4	LYS	187	Clr4	LYS	205	Hinge-SET	21 Å
Clr4	LYS	187	Clr4	LYS	472	Hinge-ARL	25 Å
Clr4	LYS	193	Clr4	LYS	211	SET-SET	26 Å
Clr4	LYS	334	Clr4	LYS	338	SET-SET	8 Å
Clr4	LYS	372	Clr4	LYS	472	SET-ARL	20 Å
Clr4	LYS	464	Clr4	LYS	472	ARL-ARL	17 Å
Clr4	LYS	464	Clr4	LYS	478	ARL-ARL	16 Å
Clr4	LYS	472	Clr4	LYS	478	ARL-ARL	19 Å
Clr4	GLY	1	H2A	LYS	95	CD-H2A	25 Å
Clr4	LYS	154	H2A	LYS	9	CD-H2A	30 Å
Clr4	LYS	154	H2A	LYS	36	CD-H2A	27 Å
Clr4	LYS	165	H2A	LYS	36	CD-H2A	32 Å

bioRxiv preprint doi: https://doi.org/10.1101/2025.08.28.672867; this version posted August 30, 2025. The copyright holder for this preprint (which was not certified by peer review) is the author/funder, who has granted bioRxiv a license to display the preprint in perpetuity. It is made available under aCC-BY 4.0 International license.

**Clr4**	LYS	187	H2A	LYS	36	CD-H2A	34 Å
**Clr4**	LYS	472	H2A	LYS	36	ARL-H2A	36 Å
**Clr4**	GLY	1	H2B	LYS	31	CD-H2B	44 Å
**Clr4**	GLY	1	H2B	LYS	105	CD-H2B	18 Å
**Clr4**	GLY	1	H2B	LYS	113	CD-H2B	27 Å
**Clr4**	GLY	1	H2B	LYS	117	CD-H2B	32 Å
**Clr4**	LYS	17	H2B	LYS	105	CD-H2B	28 Å
**Clr4**	LYS	17	H2B	LYS	117	CD-H2B	36 Å
**Clr4**	LYS	25	H2B	LYS	105	CD-H2B	31 Å
**Clr4**	LYS	109	H2B	LYS	105	Hinge-H2B	32 Å
**Clr4**	LYS	113	H2B	LYS	117	Hinge -H2B	25 Å
**Clr4**	LYS	114	H2B	LYS	8	Hinge -H2B	56 Å
**Clr4**	LYS	122	H2B	LYS	113	Hinge -H2B	24 Å
**Clr4**	LYS	122	H2B	LYS	117	Hinge -H2B	26 Å
**Clr4**	LYS	154	H2B	LYS	117	Hinge -H2B	15 Å
**Clr4**	LYS	154	H2B	LYS	122	Hinge -H2B	20 Å
**Clr4**	LYS	174	H2B	ALA	1	Hinge -H2B	25 Å
**Clr4**	LYS	180	H2B	ALA	1	Hinge -H2B	13 Å
**Clr4**	LYS	187	H2B	ALA	1	Hinge -H2B	23 Å
**Clr4**	LYS	464	H2B	ALA	1	ARL-H2B	45 Å
**Clr4**	GLY	1	H3	LYS	4	CD-H3	30 Å
**Clr4**	GLY	1	H3	LYS	18	CD-H3	10 Å
**Clr4**	GLY	1	H3	LYS	23	CD-H3	11 Å
**Clr4**	GLY	1	H3	LYS	27	CD-H3	21 Å
**Clr4**	GLY	1	H3	LYS	56	CD-H3	42 Å
**Clr4**	GLY	1	H3	LYS	79	CD-H3	15 Å
**Clr4**	LYS	17	H3	LYS	4	CD-H3	24 Å
**Clr4**	LYS	17	H3	LYS	27	CD-H3	31 Å
**Clr4**	LYS	25	H3	LYS	27	CD-H3	29 Å
**Clr4**	LYS	94	H3	LYS	4	Hinge-H3	19 Å
**Clr4**	LYS	127	H3	LYS	27	Hinge-H3	31 Å
**Clr4**	LYS	154	H3	LYS	23	Hinge-H3	32 Å
**Clr4**	LYS	154	H3	LYS	122	Hinge-H3	45 Å
**Clr4**	LYS	211	H3	LYS	4	SET-H3	37 Å
**Clr4**	LYS	338	H3	LYS	4	SET-H3	27 Å
**Clr4**	LYS	372	H3	LYS	4	SET-H3	4 Å
**Clr4**	LYS	464	H3	LYS	4	ARL-H3	24 Å
**Clr4**	LYS	464	H3	LYS	18	ARL-H3	17 Å
**Clr4**	LYS	464	H3	LYS	23	ARL-H3	16 Å
**Clr4**	LYS	464	H3	LYS	27	ARL-H3	21 Å
**Clr4**	LYS	472	H3	LYS	4	ARL-H3	18 Å
**Clr4**	LYS	472	H3	LYS	27	ARL-H3	38 Å
**Clr4**	LYS	472	H3	LYS	122	ARL-H3	32 Å
**Clr4**	GLY	1	H4	LYS	20	CD-H4	15 Å
**Clr4**	GLY	1	H4	LYS	31	CD-H4	30 Å
**Clr4**	GLY	1	H4	LYS	91	CD-H4	31 Å

bioRxiv preprint doi: https://doi.org/10.1101/2025.08.28.672867; this version posted August 30, 2025. The copyright holder for this preprint (which was not certified by peer review) is the author/funder, who has granted bioRxiv a license to display the preprint in perpetuity. It is made available under aCC-BY 4.0 International license.

**Clr4**	LYS	17	H4	LYS	20	CD-H4	31 Å
**Clr4**	GLY	1	Ub	LYS	6	CD-Ub	8 Å
**Clr4**	GLY	1	Ub	LYS	48	CD-Ub	19 Å
**Clr4**	LYS	17	Ub	LYS	48	CD-Ub	12 Å
**Clr4**	LYS	154	Ub	LYS	48	Hinge-Ub	31 Å
**Clr4**	LYS	472	Ub	LYS	6	ARL-Ub	24 Å

Table S2: List of *S. pombe* strains used in this study.

Strain #	Genotype	Source
**SPY3**	*mat1Msmto leu1-32 his2 L(Bgl II)::ade6+ ade6 DN/N*	S.I. Grewal
**SPY7283**	*SPY3 hphR-5'(1kb)-3xFlag-clr4+ #1*	Lab stock
**SPY7305**	*SPY3 clr4Δ::kanR #1*	Lab stock
**SPY11057**	*SPY3 clr4 all KR #1*	this study
**SPY11058**	*SPY3 clr4 all KR except K455/K472 #1*	this study
**SPY11059**	*SPY3 clr4 1st half KR #2*	this study
**SPY11071**	*SPY3 hphR-Flag-clr4 2nd half KR #7*	this study
**SPY10562**	*SPY3 hphR-FLAG-Clr4-2ndhalf-KtoR_except K455/K472*	this study
**SPY5071**	*h- leu1-32 ade6-M210 ura4Δ::10XTetO-ade6 #1*	Lab stock
**SPY5086**	*h- leu1-32 ade6+-M210 ura4Δ::10XTetO- ade6+ clr4Δ::natMX6-Pclr4-TetR-2XFLAG-clr4-I epe1Δ::kanMX6*	Lab stock
**SPY10655**	*h- leu1-32 ade6-M210 ura4Δ::10XTetO-ade6 clr4Δ:nat-clr4p-TetR-Clr4-allKtoR-except K455,K472-ΔCD4*	this study
**SPY10657**	*h- leu1-32 ade6-M210 ura4Δ::10XTetO-ade6 clr4Δ:nat-clr4p-TetR-Clr4-1st half-KtoR-ΔCD*	this study
**SPY138**	*h+ leu1-32 ade6-M210 ura4-D18 otr1R(SphI)::ade6+*	K. Ekwall
**SPY8328**	*SPY138 clr4Δ::ura4-KAN #1*	Lab stock
**SPY5233**	*SPY138 Kan-1kb5'UTR-3xFlag-clr4 #1*	Lab stock
**SPY11061**	*SPY138 hphR-Flag-clr4 all KR #21*	this study
**SPY11063**	*SPY138 hphR-Flag-clr4 all KR except K455/K472*	this study
**SPY11065**	*SPY138 hphR-Flag-clr4 1st half KR*	this study

bioRxiv preprint doi: https://doi.org/10.1101/2025.08.28.672867; this version posted August 30, 2025. The copyright holder for this preprint (which was not certified by peer review) is the author/funder, who has granted bioRxiv a license to display the preprint in perpetuity. It is made available under aCC-BY 4.0 International license.

**SPY11067**	*SPY138 hphR-Flag-clr4 2nd half KR*	this study
**SPY11277**	*h+ leu1-32 ade6-M210 ura4DS/E otr1R(SphI)::ura4+ oriA FLAG-clr4 Rik1-9myc cul4-GFP*	this study
**SPY11278**	*h+ leu1-32 ade6-210 ura5-D18 otr1R(SPhl)::ade6+ 3xflag-clr4, Rik1-9myc::kan, cul4680R::Nat*	this study
**SPY1702**	*6079 h3.2-K14R h3.1/h4.1 ::his3+ h3.3/h4.3 ::arg3+ ade6-210 otr1R(Sphl):ade6+*	R. Allshire
**SPY1700**	*6076 h3.2-K14A h3.1/h4.1 ::his3+ h3.3/h4.3 ::arg3+ ade6-210 otr1R(Sphl):ade6+*	R. Allshire
**SPY10829**	*h+ h3.2-K14R h3.1/h4.1::his3+ h3.3/h4.3::arg3+ Kan-1kb5'UTR-3xFlag-Clr4 ade6-M210 otr1R(Sphl):ade6+*	this study
**SPY10825**	*h+ h3.2-K14A h3.1/h4.1::his3+ h3.3/h4.3::arg3+ Kan-1kb5'UTR-3xFlag-Clr4 ade6-M210 otr1R(Sphl):ade6+*	this study
**SPY137**	*h+ leu1-32 ade6-M210 ura4DS/E otr1R(SphI)::ura4+ oriA*	K. Ekwall
**SPY815**	*SPY137 clr4Δ::kanR*	Hong et al.

Table S3: List of plasmids used in this study.

Plasmid #	Description	Source
**pDM2113**	pGEX-6P-1-Clr4 WT	Lab stock
**pDM2142**	pGEX-6P-1-Clr4-Y451N	Lab stock
**pDM1000**	pGEX-6P-1-Clr4 ΔCD (aa 70 - 490)	Lab stock
**pDM1906**	pGEX-6P-1-Clr4 SET (aa 192-490)	Lab stock
**pDM2499**	pGEX-6P-1-Clr4 ΔHinge (Δaa 70-191) 2×GSS	This study
**pDM2426**	pGEX-6P-1-Clr4 ΔSET (aa 1-192)	This study
**pDM2432**	pGEX-6P-1-Clr4 ΔhingeΔSET (aa 1-69)	This study
**pDM2428**	pGEX-6P-1-Clr4N-Set2	This study
**pDM2132**	pGEX-6P-1-Clr4-K455,472R	Lab stock
**pDM2433**	pGEX-6P-1-Clr4-W31G	This study
**pDM2451**	pGEX-6P-1-Clr4-W31G,Y451N	This study
**pDM2458**	pREP1-nmt1-LEU2 6×His-Ub	Lab stock
**pDM2455**	438-A	L. Farnung
**pDM2456**	438-C	L. Farnung
**pDM2457**	pbiGBac	L. Farnung
**pDM844**	pET3a-H2A, *Xenopus laevis*	Lab stock
**pDM845**	pET3a-H2B, *Xenopus laevis*	Lab stock
**pDM1363**	pET3a-H3 C110A, *Xenopus laevis*	Lab stock

bioRxiv preprint doi: https://doi.org/10.1101/2025.08.28.672867; this version posted August 30, 2025. The copyright holder for this preprint (which was not certified by peer review) is the author/funder, who has granted bioRxiv a license to display the preprint in perpetuity. It is made available under aCC-BY 4.0 International license.

**pDM1365**	pET3a-H3 K9C,C110A, *Xenopus laevis*	Lab stock
**pDM2452**	pET3a-H3 K14R,C110A, *Xenopus laevis*	This study
**pDM2453**	pET3a-H3 K9M,C110A, *Xenopus laevis*	This study
**pDM2454**	pET3a-H3K9M,K14R,C110A, *Xenopus laevis*	This study
**pDM847**	pET3a-H4, *Xenopus laevis*	Lab stock

Table S4: List of qPCR primers used in this study.

Primer	Sequence
**dg_F_AS131**	AAGGAATGTGCCTCGTCAAATT
**dg_R_AS132**	TGCTTCACGGTATTTTTTGAAATC
**dh_F_AS133**	GTATTTGGATTCCATCGGTACTATGG
**dh_R_AS134**	ACTACATCGACACAGAAAA-GAAAACAA
**act1_F_AS49**	CAACCCTCAGCTTTGGGTCTTG
**act1_R_AS50**	TCCTTTTGCATACGATCGGCAATAC
**mat2P_F_NI880**	GTCTCCTAACGTCCTGATAATG
**mat2P_R_NI881**	CTTCAGCCAAATGCTCAATAAT
**tlh1_F_NI882**	CTGGGCGGTAGTGAAATG
**tlh1_R_NI883**	AAGAACAAGGACGAGGTAAAG
**fbp1_F_GJ173**	ATTGACGCCGGTGTTAGTGTAGGT
**fbp1_R_GJ174**	TGACACGATGACCTGTGGTAAGCA
